# Myeloid Cell CK2 Regulates Inflammation and Resistance to Bacterial Infection

**DOI:** 10.3389/fimmu.2020.590266

**Published:** 2020-12-08

**Authors:** Sandy R. Larson, Nikki Bortell, Alysha Illies, William J. Crisler, Jennifer L. Matsuda, Laurel L. Lenz

**Affiliations:** ^1^ Immunology and Microbiology Department, University of Colorado School of Medicine, Aurora, CO, United States; ^2^ Department of Biomedical Research, National Jewish Health, Denver, CO, United States

**Keywords:** kinase, CK2, myeloid cells, monocyte, *Listeria monocytogenes*, infection, innate immunity, immune regulation

## Abstract

Kinase activity plays an essential role in the regulation of immune cell defenses against pathogens. The protein kinase CK2 (formerly casein kinase II) is an evolutionarily conserved kinase with hundreds of identified substrates. CK2 is ubiquitously expressed in somatic and immune cells, but the roles of CK2 in regulation of immune cell function remain largely elusive. This reflects the essential role of CK2 in organismal development and limited prior work with conditional CK2 mutant murine models. Here, we generated mice with a conditional (floxed) allele of *Csnk2a*, which encodes the catalytic CK2α subunit of CK2. When crossed to *Lyz2*-cre mice, excision of *Csnk2a* sequence impaired CK2α expression in myeloid cells but failed to detectably alter myeloid cell development. By contrast, deficiency for CK2α increased inflammatory myeloid cell recruitment, activation, and resistance following systemic *Listeria monocytogenes* (Lm) infection. Results from mixed chimera experiments indicated that CK2α deficiency in only a subset of myeloid cells was not sufficient to reduce bacterial burdens. Nor did cell-intrinsic deficiency for CK2α suffice to alter accumulation or activation of monocytes and neutrophils in infected tissues. These data suggest that CK2α expression by *Lyz2*-expressing cells promotes inflammatory and anti-bacterial responses through effects *in trans*. Our results highlight previously undescribed suppressive effects of CK2 activity on inflammatory myeloid cell responses and illustrate that cell-extrinsic effects of CK2 can shape inflammatory and protective innate immune responses.

## Introduction

Reversable phosphorylation is a key mechanism for regulation of nearly all cellular processes, including cell growth, survival, development, and differentiation. The protein kinase CK2 (formerly casein kinase II) is ubiquitously expressed and highly conserved through evolution. CK2 protein can be detected within nearly every cellular compartment—including the plasma membrane, cytosol, cytoskeleton and the nucleus (1). CK2 is a tetrameric holoenzyme with the reported ability to phosphorylate serine/threonine as well as tyrosine residues (2). Inhibition of CK2 has further been associated with modulation of diseases ranging from infections to sterile inflammatory disorders, cancer, chronic colitis, ischemia, multiple sclerosis, and glomerulonephritis (3–7). Due to its upregulation in numerous cancers, CK2 inhibitors have been investigated in multiple clinical trials (5, 8, 9). Inhibition and other approaches have identified hundreds of putative CK2 targets and implicated CK2 kinase activity in diverse aspects of cellular function—including the function of immune cells (2, 10, 11). A better understanding of how CK2 impacts immune responses and immune cell function could reveal strategies to selectively target CK2 for improvement of immune responses and treatment of infectious and other diseases.

Previous studies using chemical inhibitors of CK2 have implicated this kinase in regulation of several signaling pathways that are important for innate immune responses, including PI3K-Akt, JAK-STAT, and NF-κB (12–18). CK2 inhibitors dampened NF-κB activation in macrophages and consequent IL-1, IL-6 and IL-10 cytokine secretion by these cells in response to LPS stimulation (19). Conversely, CK2 inhibition increased p38-MAPK and JNK signaling pathways (14, 19). In viral infection settings, CK2 inhibition reportedly boosts type I interferon (IFN) production in macrophages. Direct phosphorylation of RIG-I, and the phosphorylation of the phosphatase PP2A by CK2 were suggested to limit TBK1/IRF3 activation (20, 21). Moreover, targeting of CK2 activity has recently been implicated in the suppression of SARS-CoV 2 viral replication (22, 23). CK2 also directly phosphorylates transcription factors including NF-κB, SP-1, and ATF3, which can either promote or hinder their binding to DNA (6, 15, 24, 25). Phosphorylation of SP-1 cooperates with NF-κB transcription, whereas CK2-ATF3 interactions downregulate NF-κB and TLR signaling (6, 26, 27). Thus, the overall impact of CK2 on immune cell signaling pathways and the overall immune response have been difficult to predict.

Efforts to better define how CK2 regulates signaling within immune cells have been further complicated by the fact that engineered mice deficient in either the catalytic α or α’ subunits of CK2, or the regulatory β subunits are non-viable or non-fertile (28, 29). Thus, most prior studies have been limited to using small-interfering RNAs or non-specific inhibitors to study CK2 kinase activity (30). Recently, mutagenesis using the Cre/loxP recombination system has been used to successfully target expression of CK2α and CK2α’ subunits in mice. However, studies to date have focused on investigations of neuronal behavior and T cell polarization during autoimmune neuroinflammation or colitis (31–33). It thus remains unclear how deficiency in CK2 might impact the function of innate immune cells and host resistance during pathogen infection.

*Listeria monocytogenes* (Lm) is a Gram-positive bacterium and intracellular pathogen that when ingested can cause severe systemic and central nervous system infections of immune compromised individuals, the elderly, neonates, and pregnant women (34). Of the major foodborne pathogens, Lm is one of the deadliest with a case-fatality rate of over 20% (35). Innate and adaptive immune responses are both involved in clearance of systemic (i.v.) Lm infection, thus this model is frequently used to study impact of immune responses on host-pathogen interactions (36, 37). Studies with the systemic Lm infection model have revealed that myeloid cells can promote or minimize bacterial replication through effects on innate immunity, cytokine production, or direct bacterial killing. Early responses by neutrophils and Ly6C+ monocytes promote host resistance to infection, whereas *Batf3*-dependent DCs increase bacterial burdens in host tissues (38–43). Macrophages also have differing roles during Lm infection, efficiently killing a large proportion of ingested bacteria but often supporting growth of bacteria once they escape the phagolysosome and enter the cytosol and modulating their traffic to T cell zones of the spleen (44, 45).

To study the impact of CK2 catalytic activity on myeloid cell responses to infection, we have engineered mice with a conditional *Csnk2a* allele. In this report, we crossed these conditionally mutant mice to mice with a *Lyz2*-cre allele to selectively deplete CK2α expression in myeloid cell populations. We found that despite substantially reduced CK2α protein in isolated macrophages from the resulting MϕCK2α^−/−^ mice myeloid cell development was not detectably altered by CK2α deficiency. However, host resistance to Lm in these mice was significantly improved as determined by reduced bacterial expansion in infected tissues. Reduced bacterial burdens in these animals correlated with increased accumulation and activation of inflammatory myeloid cells, particularly in the spleen. However, cell-intrinsic loss of CK2α did not provide a competitive advantage for cells to amass and/or become activated. Rather, mixed chimera studies suggested that the effects of CK2α on myeloid cell recruitment were likely due to the absence of CK2α signaling in an extrinsic myeloid cell population. Together, these studies provide the first direct evidence that myeloid cell CK2 regulates inflammatory and innate immune responses in an *in vivo* infectious bacterial setting and suggest that these effects are due to cell extrinsic CK2 activity.

## Materials and Methods

### Mice

All animal studies were conducted with approval by the Animal Care and Use Committees at the University of Colorado School of Medicine and National Jewish Health. C57BL/6J and B6.*Lyz2* ^cre^ (LysM^cre^) mice were purchased through Jackson Laboratories and maintained in our specific pathogen free colonies at University of Colorado Office of Laboratory Animal Resources. Experiments used both male and female mice that were aged (8–12 weeks) and sex matched. No significant differences were noted in responses of male and female mice. The *Csnk2a* targeting construct was obtained from KOMP/EUCOMM. The construct was electroporated into B6/N ES cells and used to generate chimeric founders by the National Jewish Health Mouse Genetics Core. The targeting vector contained *loxP* sites flanking the third exon of the *Csnk2a1* gene and *FRT* sites flanking the IRES-LacZ/neomycin selection cassette. Chimeric mice were thus first crossed to mice expressing FLPo recombinase to excise the LacZ reporter and neomycin selection cassette. Offspring were screened using primers specific for the transgene construct: 5’ - CTATGTAGCTGAGGGTGACCTTGAG and 5’ - CAGCCTGGGCTACAGATGACAGATG. MϕCK2α^−/−^ mice were generated by crossing B6.LysM^cre^ with CK2α^fl/fl^ mice.

### Bacterial Infections

WT mouse-passaged *L. monocytogenes* [Lm; strain 10403s from D. Portnoy (46)] were thawed from frozen stocks and diluted for growth to log phase in tryptic soy broth (TSB) (MP Biomedicals) and 50 μg/ml streptomycin (Sigma). Log phase bacteria were then diluted in PBS and injected i.v. in the lateral tail vein. Mice received a single sublethal dose of 10e+4 colony forming units (CFUs). At indicated times points, tissues were cut in half and weighed for either CFU determination or cell collection for flow cytometry. For determination of CFUs, tissues were harvested into 0.02% NP-40, homogenized for 45 s with a tissue homogenizer (IKA Works, Inc.). Serial dilutions were plated on TSB + streptomycin (50 μg/ml) agar plates and grown overnight at 37°C. To obtain BMDMs, bone marrow was flushed from mice femurs and tibias and cultured for 6 days in BM macrophage media (DMEM, 10% FBS, 1% sodium pyruvate, 1% L-glutamine, 1% 2-Mercaptoethanol, 10% L-cell conditioned media). Fresh media was added on day 3. On day 6, 2e+6 cells per well were plated in 24 well plates using antibiotic-free media. The next morning, 2e+5 log phase Lm were added to each well. Cells were washed and incubated in media containing 10 μg/ml gentamicin until harvest, at which time media was aspirated and cells were lysed in 0.5 ml of sterile 0.1% NP40 (IGEPAL). Aliquots were plated and counted as above.

### Tissue Processing

Spleens and livers were harvested into media containing 1mg/ml collagenase type IV (Worthington) in HBSS plus cations (Gibco) and incubated for 25 min at 37°C. Spleens were then passed through a 70 uM cell strainer and treated with RBC lysis buffer (0.15 M NH_4_Cl, 10 mM KHCO_3_, 0.1 mM Na_2_EDTA, pH 7.4) for 6 mins. Livers were then re-suspended in 40% Percoll (GE Healthcare) in HBSS minus cations and underlayed with 60% Percoll. After centrifugation, liver cells were then collected from the gradient interface and underwent RBC lysis.

### Generation of Chimeric Mice

Mice received a split dose of 500 rads cesium irradiation. For bone marrow cell transfers, each host mouse received 2e+6 donor cells delivered by intravenous (i.v.) injection in a total volume of 200 μl PBS. Mice were allowed to reconstitute for 6 weeks before infection with 10e+4 Lm i.v. Reconstitution of the hematopoietic system by donor-derived cells was confirmed by staining with CD45.1/2 and flow cytometric analysis prior to infection.

### Immunoblots

BMDMs were obtained as above. Peritoneal cells were obtained by injecting 5 ml PBS with 5% FBS into the peritoneum, and then massaged to dislodge cells. Fluid was then collected with needle syringe and confirmed for the absence of blood contamination. Total cell lysates from 1e+6 cells were obtained by adding 0.02% NP-40 supplemented with Halt Protease Inhibitor Cocktail (Thermo Scientific) and 1X SDS-PAGE buffer (0.0625M Tris-Cl pH 6.8, 2% sodium dodecyl sulfate [SDS], 10% glycerol, 5% 2-Mercaptoethanol, 0.01% bromophenol blue) and loaded into 10% SDS-polyacrylamide gels. Gels were transferred onto a polyvinylidene difluoride (PVDF) membrane (Millipore) and probed for CK2α (Biomatik) and β-Actin (8H10D10; Cell Signaling), followed by secondary goat anti-rabbit IR 800 (926-32211; LI-COR) and goat anti-mouse IR 680 (926-68070; LI-COR). Blots were imaged on an Odyssey CLX (LI-COR). Expression of CK2α was normalized to β-Actin using Image Studio Lite ver5.2 (LI-COR) software.

### Phagocytosis Assays

pHrodo Green *S. aureus* Bioparticles (ThermoFisher) were reconstituted as per manufacturer’s instructions to a concentration of 1 mg/ml. The Bioparticles or Fluoresbrite YG 0.5 μM microspheres (Polysciences) were added at a 1:5, or 1:300 ratio, respectively to 1+e6 splenocytes and incubated at 37°C for 1 h. Phagocytosis was stopped by immediately washing cells in ice-cold FACS buffer (1% BSA, 0.01% NaN3, PBS) and placed on ice for further staining.

### Flow Cytometry

Prior to surface staining, anti-CD16/32 (2.4G2 hybridoma supernatant) was added to block Fc receptors Following steps were performed in FACS buffer (1% BSA, 0.01% NaN3, PBS). Fluorophore-labeled antibodies included anti-B220 (47-0452-82), CD3 (145 2C11), CD11b (M1/70), CD11c (N418), CD16/32 (93), CD34 (RAM34), CD45.1 (A20), CD45.2 (104), CD117 (2B8), F4/80 (BM8), GR-1 (RB6-8C5), Ly6C (HK1.4), Ly6G (1A8), MHCII (M5/114.15.2), NK1.1 (PK136), Sca-1 (D7), and TER-119 were purchased from either eBioScience or BioLegend. After surface staining, cells were fixed in 4% paraformaldehyde for analysis. Samples were collected using LSRFortessa (BD Biosciences) and analyzed using FlowJo software (Treestar). For Annexin V staining, Annexin V Apoptosis Detection Kit (Invitrogen) was used per manufacturer’s instructions.

### Statistical Analysis

All experiments were repeated at least three time, unless otherwise noted. Statistical methods were performed using GraphPad Prism (La Jolla, CA, USA) software. Significance was determined by two-tailed Students t-test. A p-value of <0.05 was considered significant. In the figures, * denotes *P* values between 0.05 and 0.01, ** denotes *P* values between 0.01 and 0.001, and *** denotes *P* values less than 0.001.

## Results

### Generation of MϕCK2α^−/−^ mice

**Figure 1:**
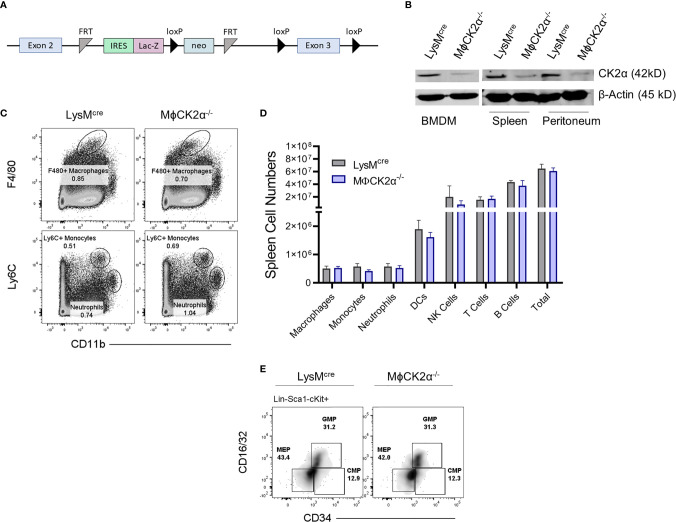
Development of myeloid cell populations is not detectably altered in MϕCK2α^−/−^ mice. **(A)** Schematic of *Csnk2a* targeting vector. **(B)** Immunoblots of lysates from BMDMs, splenocytes, or peritoneal cavity cells of LysM^cre^ or LysM^cre^CK2α^fl/fl^ (MϕCK2α**^−^** ^/^ **^−^**) mice were probed for CK2α or β-actin as a loading control. n = 2. **(C)** Splenic myeloid cell populations were stained and analyzed by flow cytometry. Plots depict gating for representative F4/80+ macrophages, Ly6C+ monocytes, and neutrophils (also Ly6G+). **(D)** Cells from spleen were stained for flow cytometry to enumerate immune cell populations. Data are from pooled experiments, n = 4–12 mice/group. **(E)** Cells from bone marrow were stained for flow cytometry to evaluate bone marrow precursor populations including common myeloid progenitors (CMPs), granulocyte-monocyte progenitors (GMPs), and megakaryocyte-erythrocyte progenitor (MEP). Cells were pre-gated on Lin-(B220, CD3, CD11b, GR-1 and TER-119), Sca-1-, cKit-. Density plots are representative from two experiments.

Global deletion of CK2α is embryonically lethal, therefore we generated a conditional knockout to investigate the function of CK2 in myeloid cells. Conditional knockout for the CK2α subunit was generated by introducing a targeting vector incorporating loxP sites that flank the third exon of the *Csnk2a1* gene and *Flp* recombinase flanking the selection cassette into B6/N ES cells (**Figure 1A**). Cells were selected by neomycin antibiotic treatment and clones used to produce chimera mice. These were then then crossed to FLP recombinase mice to delete the selection cassette. To study specific CK2α deletion in myeloid cells, these mice were then crossed to LysM^cre^ expressing mice to generate MϕCK2α^−/−^ mice (1). Progeny are viable and fertile, with no observable development impairments compared to littermate controls. Reduction of CK2α expression was confirmed in bone marrow-derived macrophages (BMDMs), as well as primary cells from the spleen and peritoneal cavity (**Figure 1B**). Both myeloid and lymphocyte cellular compartments within MϕCK2α^−/−^ mice were maintained. Of importance, naïve LysM^cre^ controls and MϕCK2α^−/−^ mice had comparable numbers of M-lysozyme expressing cells including F4/80+ macrophages, Ly6C+ monocytes and neutrophils (**Figures 1C, D** **)**. Further, we evaluated the bone marrow compartments that ultimately give rise to granulocytes and monocytes—the multipotent common myeloid progenitor (CMP) and granulocyte-monocyte progenitor (GMP). The proportions of CMP and GMP in bone marrow were comparable in the LysM^cre^ controls and MϕCK2α^−/−^ mice (**Figure 1E**).

### Resistance to Systemic Lm Infection Is Increased in MϕCK2α^−/−^ Mice

**Figure 2:**
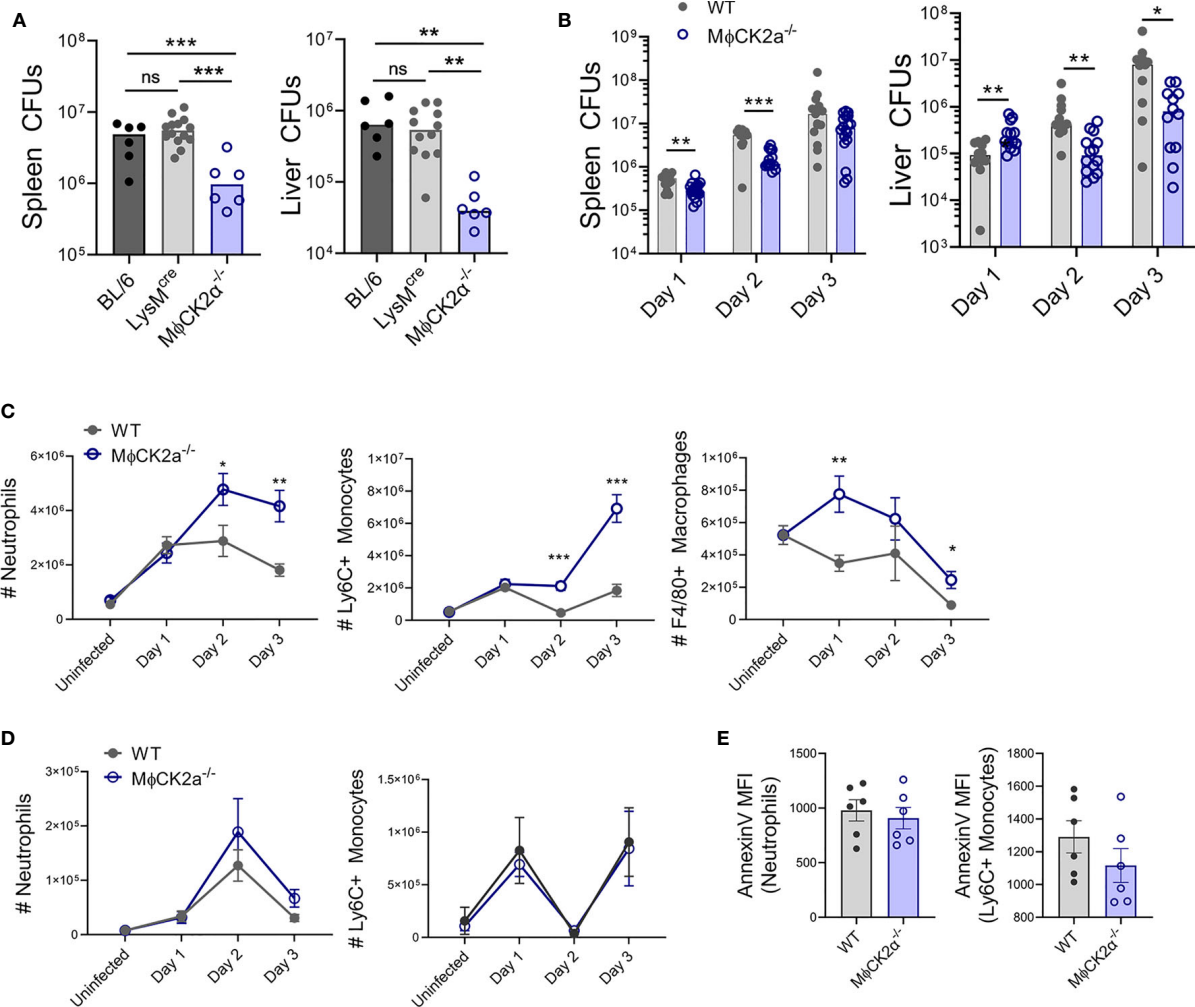
Resistance to systemic Lm infection is improved in MϕCK2α^−/−^ mice, which correlates with increased accumulation of inflammatory cells. **(A)** Lm burdens from infected C57BL/6 (B6), LysM^cre^, or MϕCK2α**^−^** ^/^ **^−^** mice. Colony forming units (CFU) per tissue were determined by dilution plating of tissue lysates at 2 days post-infection (dpi) with 10e+4 Lm. Symbols represent individual mice and bars indicate mean values. Data are pooled from two experiments and with n = 6–12 mice/group. **(B)** CFUs from BL/6 (WT) and MϕCK2α**^−^** ^/^ **^−^** mice at indicated dpi. Data pooled from three experiments, n = 9–12 mice/group. Cells were harvested from collagenase-digested **(C)** spleens and **(D)** livers of uninfected WT or MϕCK2α**^−^** ^/^ **^−^** mice or at the indicated day after Lm-infection. Myeloid cells were analyzed by flow cytometry with gating as in **Figure 1**. Mean ± SEM cell counts per organ are shown. Data are pooled from three experiments, n = 9–12 mice/group. **(E)** Splenocytes harvested 2 dpi from infected WT and MϕCK2α**^−^** ^/^ **^−^** mice were stained for surface Annexin V to assess apoptosis. Shown are staining intensity (geometric MFI) for Annexin V stains on gated neutrophils and monocytes. Data pooled from two experiments, n = 6 mice/group. *p < 0.05, **p < 0.01, ***p < 0.001 and ns = p < 0.05 as measured by Student t-test.

Given the apparently normal development of myeloid cells in MϕCK2α^−/−^ mice, we infected groups of BL/6, LysM^cre^, and MϕCK2α^−/−^ mice with a challenge dose of Lm (10^4^ cfu i.v.) to investigate if CK2α expression might modulate innate host defense against systemic bacterial infection. Previous reports had suggested that chemical inhibitors of CK2α increase type 1 interferon signaling in cultured cells (2, 3). Myeloid cells are a dominant source of type I IFNs following systemic Lm infection but this type I IFN is detrimental for resistance as early as 3–4 dpi after systemic Lm infection, due in part to the impaired activation of myeloid cells by IFNγ (4–9). Thus, we expected MϕCK2α*^−^* ^/^ *^−^* mice to show elevated susceptibility to Lm. It was thus surprising to observe that bacterial burdens (colony forming units; cfu) were significantly lower in both spleen and liver of the MϕCK2α^−/−^ mice (5.7- and 15.2-fold decrease, respectively) as early as 2 days of infection (**Figure 2A**). Lm burdens were comparable between control BL/6 and LysM^cre^ mice, indicating the cre insertion in to the *Lyz2* locus did not alter host resistance in this infection setting. Protective effects of MϕCK2α-deficiency were observed even at 1 day post-infection (dpi) in the spleens of MϕCK2α^−/−^ mice (**Figure 2B**). However, at this early time point bacterial burdens in the liver were instead slightly elevated. This may be due to increased early uptake or survival of the bacteria in the CK2a-deficient livers. Control of Lm burdens continued to be significantly better in both the livers and spleens at least until 3 dpi (**Figure 2B**). Hence, the loss of CK2α expression in myeloid cells improved the ability of mice to resist Lm at early stages after a systemic infection.

### CK2α Attenuates the Accumulation of Inflammatory Myeloid Cells

Given expression of LysM in monocytes and neutrophils, we hypothesized that the improved resistance of MϕCK2α^−/−^ mice might reflect improved ability of these inflammatory cells to respond to the Lm infection. Consistent with this hypothesis, accumulation of neutrophils and Ly6C+ monocytes in spleens of infected MϕCK2α^−/−^ mice was significantly greater than that seen in WT mice beginning at 2 dpi (4.77e+6 and 2.11e+6 *versus* 2.88e+6 and 4.65e+5 cells) (**Figure 2C**). Additionally, numbers of splenic F4/80+ macrophages were robustly increased at 1 dpi (7.75e+5 in MϕCK2α^−/−^ mice *versus* 3.43e+5 in WT). By contrast, the reduced bacterial burdens in livers of MϕCK2α^−/−^ mice were not associated with significant increases in the number of inflammatory myeloid cells recovered from this organ (**Figure 2D**). Very few F4/80+ macrophages were recovered from the livers and thus were not quantified. It is possible that the failure to observe differences in myeloid cell populations in the liver in part reflect the low yield or difficulty in recovering these cells from this tissue. Regardless, our results here indicated that at least in the spleen myeloid cell responses are significantly suppressed by CK2 expression in a Lyz2+ cell population(s).

CK2 is an important regulator of the cell cycle (10), thus we considered that the accumulation of cells in the MϕCK2α^−/−^ mice might reflect altered cell survival. To estimate the prevalence of apoptosis in myeloid cells, we measured surface staining with Annexin V on splenocytes from infected mice. At 2 dpi, fluorescence intensity of annexinV staining was not significantly different on the monocytes from WT and MϕCK2α^−/−^ mice (**Figure 2E**). There was also no difference in annexin V staining on neutrophils from the WT and MϕCK2α^−/−^ spleens. These data suggest the increased numbers of inflammatory cells in the MϕCK2α^−/−^ spleens are not due to effects of CK2 activity on survival of these inflammatory cells. We also failed to observe any difference in the rate at which bone marrow-derived macrophages cultured from WT and MϕCK2α^−/−^ mice became confluent, suggesting CK2α expression does not curtail myeloid cell proliferation. Thus, future studies will be necessary to specifically address if altered chemotaxis to or exit from infected tissues accounts for the increased accumulation of myeloid cells in the infected MϕCK2α^−/−^ mice as well as the precise mechanism(s) by which CK2α modulates these processes.

### CK2α Attenuates the Activation and Bactericidal Potential of Inflammatory Myeloid Cells

**Figure 3:**
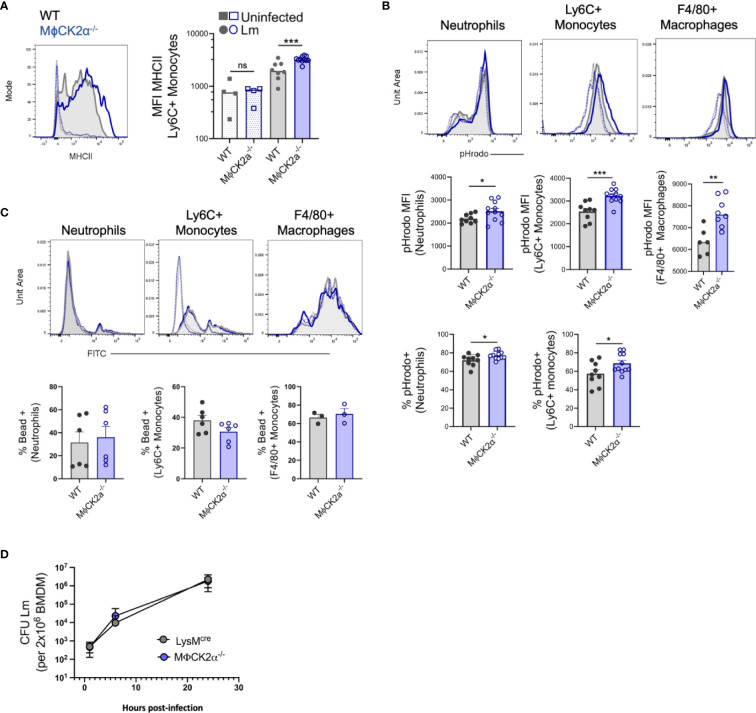
Enhanced activation of inflammatory myeloid cells from MϕCK2α^−/−^ mice. Splenocytes were harvested from uninfected and Lm infected WT or MϕCK2α**^−^** ^/^ **^−^** mice and **(A)** surface MHC II was evaluated on splenic Ly6C+ monocytes 2 dpi using flow cytometry. Histogram shows MHCII staining intensity on cells from uninfected (open, dotted line) and Lm-infected (solid line) WT (gray) and MϕCK2α**^−^** ^/^ **^−^** (blue) mice. Data are pooled from two experiments, n = 4–8 mice/group. **(B)** Splenocytes at 2 dpi were cultured 1 h with pHrodo particles. Fluorescence intensity of particles was evaluated on the indicated gated cells. Top histogram plots indicate fluorescence for WT (gray, filled line) and MϕCK2α**^−^** ^/^ **^−^** (blue, open line) cells. Uninfected samples are of corresponding color (dotted line). Middle graphs depict quantification of pHrodo MFI. Bottom graphs enumerate the percentage of cells positive for pHrodo, as determined by -pHrodo control. Data are pooled from two to three experiments, n = 3–12 mice/group. **(C)** FITC fluorescence intensity is shown in top histogram panels. Uninfected (open) and Lm-infected (filled), WT (gray), and MϕCK2α**^−^** ^/^ **^−^** (blue) are overlaid. Below graphs quantify the percentage of FITC (bead) positive as determined by -bead controls. Data are pooled from one to two experiments, n = 6 mice/group. **(D)** Lm replication in bone marrow-derived macrophages (BMDMs) cultivated from WT (gray) and MϕCK2α**^−^** ^/^ **^−^** (blue) mice. BMDMs were plated 2 × 10e+6 per well and infected with 2 × 10e+5 live Lm. Shown is one of two experiments using BMDMs from two to three individual mice per genotype per experiment. *p < 0.05, **p < 0.01, ***p < 0.001 and ns = p < 0.05 as measured by students t-test.

We further assessed activation of the control and CK2α-deficient myeloid cells accumulating in tissues of Lm-infected mice. Myeloid cell surface MHCII was modestly elevated on the splenic MϕCK2α^−/−^ Ly6C+ monocytes (**Figure 3A**). This suggested that CK2 expression in monocytes might normally act to dampen bactericidal activation in response to IFNγ stimulation. To more directly evaluate their bactericidal potential, splenocytes from infected mice were incubated with *Staphylococcus aureus* bioparticles labeled with the pH-sensitive dye (pHrodo). These experiments revealed that both the overall MFI and the proportion of pHrodo positive neutrophils and monocytes was significantly higher in the MϕCK2α^−/−^ cells (**Figure 3B**). Nearly all splenic F4/80+ macrophages were pHrodo positive in both groups of mice. However, the mean fluorescence of the pHrodo staining was significantly increased in the MϕCK2α^−/−^ mice. To determine if the increased pHrodo staining in MϕCK2α^−/−^ cells might be attributable to increased particle uptake, we incubated splenocytes from infected MϕCK2α^−/−^ and WT mice with fluorescently labeled microbeads. Regardless if neutrophils and F4/80+ macrophages were deficient for CK2α or derived from Lm infected mice, we failed to observe any enhancement in their endocytic capacity (**Figure 3C**). Ly6C+ monocytes from the Lm infected mice did demonstrate enhanced bead uptake, but this was true regardless of CK2α expression. Thus, while expression of CK2α did not detectably alter particle uptake by myeloid cells from the infected animals its expression significantly impaired phagosome maturation as indicated by the reduced pHrodo fluorescence in the WT cells. Together with the data above, these findings argue that the improved resistance of MϕCK2α^−/−^ mice reflects improved myeloid cell recruitment and activation. Interestingly, we did not observe any defect in the ability of macrophages cultivated from the bone marrow of WT and MϕCK2α^−/−^ mice to support growth of Lm (**Figure 3D**). This result indicates that absent any external stimulation CK2 failed to impact Lm survival in these cells. Thus, CK2 may regulate myeloid cell responses to specific stimuli encountered *in vivo* or may play a unique role(s) in dampening responsiveness/antimicrobial function of a specific myeloid cell population encountered in the context of systemic bacterial infection.

### Cell-Intrinsic CK2α Is Not Sufficient to Alter Myeloid Cell Accumulation or Activation in Infected Tissues

**Figure 4:**
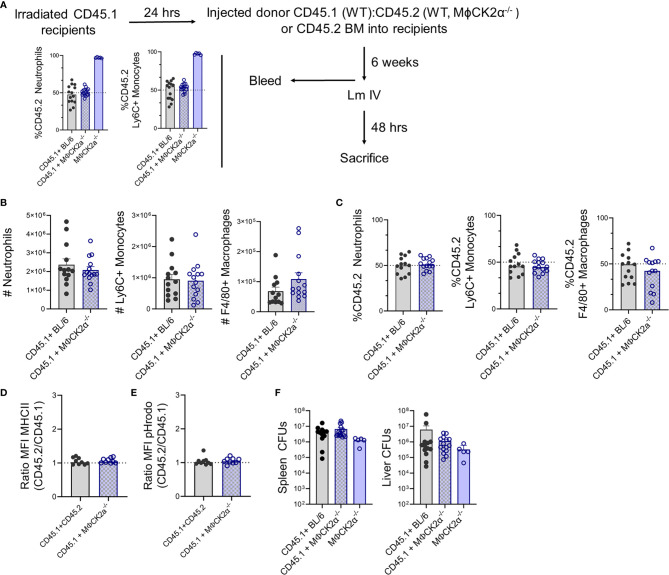
CK2α suppresses myeloid cell accumulation for increased Lm burdens *in trans*. Bone marrow (BM) chimera experiments. **(A)** Schematic of experiment and graphs summarizing % of CD45.2 neutrophils and Ly6C+ monocytes in blood of irradiated CD45.1 recipients at 6 weeks after reconstitution with 1:1 mixtures of WT CD45.1 plus CD45.2 WT or MϕCK2α**^−^** ^/^ **^−^** BM cells. The proportion of CD45.2+ cells is also shown for mice reconstituted with only CD45.2 MϕCK2α**^−^** ^/^ **^−^** donor cells. **(B–E)** Splenocytes were harvested from Lm infected CD45.1+BL/6 and CD45.1+ MϕCK2α**^−^** ^/^ **^−^** chimeras 2 dpi. **(B)** Total numbers of neutrophils, Ly6C+ monocytes, and F4/80+ macrophages in the mixed chimeras were enumerated by cell counts and flow cytometry. **(C)** Proportions of CD45.2+ neutrophils, Ly6C+ monocytes, and F4/80+ macrophages in the infected mixed chimeric animals. Surface expression of **(D)** MHCII and **(E)** fluorescence of pHrodo at 1 h after uptake were compared between CD45.1+ and CD45.2+ Ly6C+ monocytes from chimeric mice. Shown are ratios of the MFI for each marker in the CD45.2 *vs* CD45.1 cells. **(F)** Bacteria burdens (CFUs) in livers and spleens of the respective chimeric animals at 2 dpi with Lm. Figure panels **(A**, **F)** show data pooled from three experiments with four to five mice/group/experiment, a group of mice received only CD45.2 MϕCK2α**^−^** ^/^ **^−^** BM cells in the third experiment. Figure panels **(B–E)** are data pooled from three experiments, eight to ten mice/group.

We hypothesized that the above alterations in myeloid cell recruitment and functionality might be due to intrinsic CK2α signaling in the inflammatory myeloid cells themselves. To evaluate this, we constructed and infected bone marrow chimeric mice in which BL/6.*Ptprc^a^* (CD45.1) mice were lethally irradiated and reconstituted with a 1:1 mixture of bone marrow from WT CD45.1 mice plus CD45.2 BL/6 or MϕCK2α^−/−^ mice (**Figure 4A**). At 6 weeks after reconstitution, naïve chimeric mice were bled to confirm that circulating neutrophils and monocytes were present at the expected 1:1 frequency of CD45.1:CD45.2. Surprisingly, following infection the total number of neutrophils (2.08+e6) and Ly6C+ monocytes (9.05+e5) were similar in the spleens of infected chimeric mice and WT mice (compare **Figures 2C** and **4B**). This contrasts with the increased numbers of both myeloid populations in spleens of the non-chimeric MϕCK2α^−/−^ animals (**Figure 2C**). Thus, the presence of WT (CK2α+) myeloid cells in the chimeric animals was sufficient to prevent increased cellular accumulation seen in mice whose entire hematopoietic compartment was derived from the MϕCK2α^−/−^ background. Furthermore, when we measured the ratio of CD45.1 to CD45.2 cells in the infected chimeric animals, we failed to observe any selective increase in the proportion of neutrophils, Ly6C+ monocytes or F4/80+ macrophages derived from the CD45.2 WT or MϕCK2α^−/−^ bone marrow (**Figure 4C**). These data demonstrated that there was no competitive advantage (nor disadvantage) associated with CK2α deficiency in the naïve or infected mice. Moreover, they show that when WT CD45.1 cells were present in the same animal, there was no longer any increase in the accumulation of either WT or MϕCK2α^−/−^ monocytes in the infected tissues. Therefore, the absence of CK2α in myeloid cells was not sufficient to intrinsically increase their trafficking or survival.

We next evaluated myeloid cell activation (MHC II expression) and bactericidal potential (pHrodo fluorescence) in the infected chimeric animals. In neither measure did we observe any significant differences. Thus, the intensity of MHCII staining and pHrodo fluorescence were equivalent in CD45.1 and CD45.2 cells regardless if the CD45.1+ cells derived from WT or MϕCK2α^−/−^ mice (**Figures 4D, E** **)**. Importantly, the bacterial burdens in the spleens and livers also proved to be similar in the mixed chimeric animals, again indicating that the presence of WT CD45.2 cells overcame the advantages of CK2a-deficiency in the CD45.1 cells (**Figure 4F**). A group of CD45.1 mice reconstituted with CD45.2 MϕCK2α^−/−^ donor cells alone was included in one experiment, the data from which is included in **Figures 4A, F**. In these mice we did observed a trend towards lower bacterial burdens, although this did not reach statistical significance.

In summary, the results from our experiments with chimeric and non-chimeric animals argue that the effects of CK2α on myeloid cell recruitment and activation are not cell intrinsic. Rather, expression of CK2α by a *Lyz2*+ cell population(s) appears to act *in trans* to suppress both accumulation and activation of inflammatory myeloid cells, rendering host tissues more susceptible to bacterial expansion during infection.

## Discussion

Previous work using inhibitors suggested that CK2 kinase activity regulates several key immune cell signaling pathways—including PI3K-Akt, JAK-STAT, and NF-κB signaling pathways upon LPS and/or IFNγ treatment (13, 19). However, interpretation of these prior studies has been complicated by the fact that CK2 is necessary for embryonic development and thus there have not been available animal models to definitively study the immunological role of CK2 (and more specifically CK2α) using an *in vivo* model system (28, 29). In this work, we have generated a novel conditional murine knockout model with which we and others can now directly evaluate the impact of the catalytic CK2alpha subunit in immune responses. The studies here specifically deleted CK2α from lysozyme-M expressing myeloid cells. Progeny mice maintained normal development and continued to successfully breed. Moreover, we failed to observe evidence of defects in the development or function of CK2α^−/−^ myeloid cells from these mice. Indeed, the studies here argue that instead the deficiency for CK2α improved the recruitment and antibacterial activation of these cells and thus that myeloid cell expression of CK2α normally acts to limit host protection—at least in the systemic Lm infection utilized here.

Lysozyme-M expressing cells, including macrophages, monocytes, and neutrophils are important contributors to host defense against Lm and the finding of increased accumulation and activation of these cells in the MϕCK2α^−/−^ mice here is consistent with the increased resistance. CK2 is reported to regulate the cell cycle (47). However, we believe increased myeloid cell accumulation reflects an increased recruitment or retention of the cells, because we did not observe differential Annexin V staining that would indicate reduced apoptosis of the CK2α-deficient cells. Nevertheless, we have not at this point directly demonstrated any increase in chemotaxis or motility of CK2α^−/−^ myeloid cells. Further, during steady-state conditions both WT and MϕCK2α^−/−^ mice had comparable distribution of myeloid bone marrow progenitors, but our data do not exclude the possibility that CK2α might regulate myelopoiesis selectively in an inflammatory setting.

Perhaps the most surprising aspect of our results was the finding that cell-intrinsic deficiency for CK2α was not sufficient to increase the accumulation or activation of myeloid cells in the bone marrow chimeric animals. In these experiments, the host mice were of wild-type origin as were 50% of the hematopoietic cells. In contrast to mice in which the majority of *Lyz2*-expressing cells lack expression of CK2α, neither the bacterial burdens nor the numbers or proportions of accumulating myeloid cells affected by this partial CK2α deficiency in the myeloid population. These results indicate that the effects of CK2α on suppression of myeloid cell recruitment and activation are dominant. Hence, expression of CK2α by a subset of inflammatory myeloid cells in the mixed chimera animals may drive their production of a soluble factor that actively suppresses accumulation and activation of neighboring or more distal CK2α^−/−^ myeloid cells. Alternatively, there may persist in the chimeric animals a population(s) of relatively radio-resistant, lysozyme M expressing cells that mediates such suppressive effects. In either case, these finding support the notion that suppression of inflammatory cell recruitment and activation by CK2α occurs *in trans* and has important consequences for host resistance to infection. Such findings shed important light on the possible effects of CK2 activity in the context of cancers, where overexpression of CK2 activity in tumors has been shown to correlate negatively with disease prognosis (4, 15, 48). Our work suggests this may in part reflect that CK2 acts to prevent the accumulation or anti-tumor activation of myeloid cells. Recent evidence also indicates that CK2 activity is induced during and promotes SARS-Cov-2 infection (22, 23). Our data here would suggest that induction of such activity in even a subset of myeloid cells could have deleterious impact on the overall myeloid cell response to this infection.

Some tissue resident macrophage populations are relatively radio-resistant, including marginal zone and metallophilic CD169+ macrophages from the spleen, embryonic derived Kupffer cells from the liver, and bone marrow macrophages (49–52). Each of these macrophage populations require liver X receptor α (LXRα) for their maintenance, but in the presence of inflammatory stimuli LXRα actually represses the bactericidal potential of these cells including the downregulation of iNOS, the cytokines IL-1β, IL-6 and the chemokines CCL2 and CCL7 (53–55). Of interest, inhibition of CK2 was shown to prevent phosphorylation of LXRα, which ultimately promoted the transcription of the neutrophil chemokine CCL24 (56). In future experiments it will be interesting to a) determine if tissue resident, radiation-resistant macrophages are involved in suppression of myeloid cell accumulation and activation during Lm infection and b) further evaluate if CK2α regulates these responses through phosphorylating LXRα.

CK2 is reported to attenuate type I interferon production in the presence of DNA and RNA virus, as well as TLR stimulus including LPS and Poly I:C (20, 21). While protective against viral pathogens, type I interferons are detrimental to host responses to intracellular bacterium including *M. tuberculosis*, *Francisella tularensis* as well as *L. monocytogenes* (57, 58). Type I interferons limit the ability of the host to clear Lm infection in part by the direct negative regulation of type II interferon signaling on myeloid cells reducing their antimicrobial responses, limiting the recruitment of critical inflammatory Ly6C+ monocytes and inducing macrophage cellular death (59–62). Secretion of IFNβ during Lm infection is also largely restricted to splenic LysM expressing cells within 24 h upon entry into the cytosol (62, 63). While we have not exhaustively characterized interferon production in MϕCK2α^−/−^ mice, our preliminary unpublished findings are consistent with the interpretation that at least in cultured myeloid cells CK2α can suppress IFNβ production in response to pattern recognition receptor (PRR) ligation. If this is also true *in vivo*, such results might indicate that CK2α initially blunts early myeloid cell activation and accumulation at sites of infection then subsequently promotes Lm expansion through increasing type I interferon signaling. A more exhaustive analysis of infection kinetics and interferon production during *in vivo* infection will be needed to test this possibility.

In summary, we have found that the protein kinase CK2 expressed in myeloid cells negatively influences host responsiveness during early systemic Lm infection. Targeting CK2 is proving to be a successful strategy in the context of cancer therapeutics, as several inhibitors including CX-4945 and CIGB-300 are enrolled in clinical trials (64). Our work has begun to lay the groundwork targeting CK2 as a potential therapeutic to pathogenic infection. With the global threat of microbial antibiotic resistance, targeting host CK2 activity is an intriguing and novel immunomodulatory mediator (65).

## Data Availability Statement

The original contributions presented in the study are included in the article/supplementary material. Further inquiries can be directed to the corresponding author.

## Ethics Statement

The animal study was reviewed and approved by University of Colorado IACUC.

## Author Contributions

SL and LL conceived the work and wrote the paper. SL, NB, AI, and WC performed experiments and collected data. JM was instrumental in development and generation of the floxed mouse strain. LL wrote grants for financial support. All authors contributed to the article and approved the submitted version.

## Funding

The studies in this paper were funded by NIH grants R01AI131662, R21AI140499, and R33AI102264 to LL.

## Conflict of Interest

The authors declare that the research was conducted in the absence of any commercial or financial relationships that could be construed as a potential conflict of interest.
